# Synergy between type 1 fimbriae expression and C3 opsonisation increases internalisation of *E. coli *by human tubular epithelial cells

**DOI:** 10.1186/1471-2180-9-64

**Published:** 2009-03-31

**Authors:** Ke Li, Wuding Zhou, Yuzhi Hong, Steven H Sacks, Neil S Sheerin

**Affiliations:** 1 Complement Laboratory, Medical Research Council (MRC) Centre for Transplantation, Guy's Hospital, King's College London, London, SE1 9RT, UK; 2 Department of Endocrinology and Metabolic Medicine, Hangzhou Hospital of Traditional Chinese Medicine, Hangzhou, 310007, PR China; 3 Institute of Cellular Medicine, Newcastle University, Newcastle upon Tyne, NE2 4HH, UK

## Abstract

**Background:**

Bacterial infection of the urinary tract is a common clinical problem with *E. coli *being the most common urinary pathogen. Bacterial uptake into epithelial cells is increasingly recognised as an important feature of infection. Bacterial virulence factors, especially fimbrial adhesins, have been conclusively shown to promote host cell invasion. Our recent study reported that C3 opsonisation markedly increases the ability of *E. coli *strain J96 to internalise into human proximal tubular epithelial cells via CD46, a complement regulatory protein expressed on host cell membrane. In this study, we further assessed whether C3-dependent internalisation by human tubular epithelial cells is a general feature of uropathogenic *E. coli *and investigated features of the bacterial phenotype that may account for any heterogeneity.

**Results:**

In 31 clinical isolates of *E. coli *tested, C3-dependent internalisation was evident in 10 isolates. Type 1 fimbriae mediated-binding is essential for C3-dependent internalisation as shown by phenotypic association, type 1 fimbrial blockade with soluble ligand (mannose) and by assessment of a type 1 fimbrial mutant.

**Conclusion:**

we propose that efficient internalisation of uropathogenic *E. coli *by the human urinary tract depends on co-operation between type 1 fimbriae-mediated adhesion and C3 receptor -ligand interaction.

## Background

Urinary tract infection (UTI) due to uropathogenic *E. coli *is a common clinical problem, estimated to affect 40–50% of women at least once in their lifetime [1]. Frequent recurrence is an important characteristic of UTI especially among young women. Up to 25% of women who experience a first UTI will develop recurrent infections within 6 months despite appropriate treatment of the initial infection [2].

Recent research has demonstrated that uro-epithelial cells from the kidney and the bladder have the capacity to internalise *E. coli *into membrane-bound vacuoles [3,4]. Inside cells *E. coli *can establish long-lived intracellular reservoirs within the bladder mucosa that serve as a source for recurrent acute infections [5,6]. *E. coli *encode a variety of virulence factors that facilitate colonisation of the urinary tract, such as fimbrial adhesins (type 1, P, S, and Dr fimbriae) and toxins (α-hemolysin and cytotoxic necrotising factor 1 (CNF1)) [7]. In addition, Uro-pathogenic strains are usually resistant to serum bactericidal activity [8]. Of the known virulence factors associated with *E. coli*, the type 1 fimbriae associated adhesin FimH, Dr family adhesins and bacterial toxin CNF1 have been shown to directly trigger and/or modulate bacterial entry into host epithelial cells [9-11].

In addition to pathogen virulence factors, complement C3 secreted by host cells also influences the ability of *E. coli *to invade cells and tissues within the urinary tract. Studies from our group have shown that mice deficient in C3 are resistant to ascending infection and complement can alter bacterial uptake by mouse proximal tubular epithelial cells (PTECs), a primary target of *E. coli *during the acute phase of pyelonephritis [12]. Recently we reported that C3 concentration in the urine rises sufficiently during renal tract infection and *E. coli *are readily opsonised by urinary C3 [13,14]. Moreover, C3 opsonisation promotes *E. coli *invasion of human uro-epithelial cells via CD46, a complement regulatory protein expressed on host cell membranes [13]. CD46 was not involved in the binding of *E. coli *to epithelial cells. Therefore, we hypothesised that other bacterial factors may be involved in C3-dependent *E. coli *internalisation.

In the present study, we examined whether C3-dependent internalisation by host uro-epithelial cells is a general feature of *E. coli *and studied features of the bacterial phenotype that may account for any heterogeneity.

## Methods

### Bacterial strains and culture

Bacteria were grown in 5 ml of static Luria-Bertani (LB) broth at 37°C for 16 hours to induce fimbrial expression prior to use in experiments. For each experiment bacterial concentration was standardised by photospectrometry at 600 nm, and colony number confirmed using serial dilutions and plating to agar plates. *E. coli *strain J96 (serotype O4: K6) was provided by Dr. R. Welch, (University of Wisconsin, Madison, USA). It is a serum resistant, haemolysin secreting *E. coli *strain that expresses both Type 1 and P fimbriae [15]. Cystitis isolate NU14 and the isogenic FimH^- ^mutant NU14-1 were provided by Dr. S. Hultgren (Washington University school of Medicine, Missouri, USA) [9]. 31 *E. coli *isolates were obtained from the Department of Microbiology, Guy's and St. Thomas' National Health Service Foundation Trust, of which, sixteen strains were isolated from urine samples of patients suffering from acute uncomplicated cystitis and fifteen isolated from blood cultures with simultaneous UTI symptoms. The urine and blood samples were spread onto blood agar and bromothymol blue agar for the isolation and identification of *E. coli*. Diagnosis of UTI was made based on clinical symptoms and more than 10^5 ^colony-forming units (c.f.u) of *E. coli *per ml of urine. Samples associated with more than one bacterial species were excluded from the study.

### Cell line and culture

The human PTEC line was a gift from Professor. L.C. Racusen (The Johns Hopkins University School of Medicine, Baltimore, USA) [16]. The cells were grown in DMEM-F12 supplemented with 5% FCS, 5 μg/ml insulin, 5 μg/ml transferin, 5 ng/ml sodium selenium, 100 U/ml penicillin and 100 μg/ml streptomycin.

### Sera and complement inactivation

Normal human serum (NHS) was obtained from 5 healthy volunteers. After collection, serum was pooled and stored at -70°C for up to 3 months. Complement activity in serum was inactivated by incubation at 56°C for 30 minutes (Heat inactivated serum, HIS). Complement inactivation was confirmed by loss of haemolytic activity using standard methodology (data not shown).

### C3 deposition on E. coli

Bacteria were opsonised as described previously [14]. Briefly, 2 × 10^8^c.f.u *E. coli *were washed and incubated in DMEM-F12 containing 5% NHS at 37°C for 30 minutes. Bacteria were washed in 10 mM EDTA to stop further complement activation. Bacterial-bound complement proteins were eluted with 4 mM sodium carbonate, 46 mM sodium bicarbonate (pH 9.2) for 2 hours at 37°C. Bacteria were removed by centrifugation. Eluted proteins were separated by 10% SDS-PAGE under reducing conditions and transferred to a Hybond-c Extera membrane (GE Healthcare UK Limited, Bucks, UK). The membrane was sequentially incubated with blocking buffer (PBS-5% milk powder) at 4°C overnight, rabbit anti-human C3c (1/1000; Dako UK Ltd, Cambridgeshire, UK), and peroxidase-conjugated goat anti-rabbit IgG (1/5000; Dako). The membrane was then developed using the ECL system (GE Healthcare UK Limited).

### Assessment of bacterial binding and internalisation

PTECs were seeded into 24 well plates and grown to confluence. Overnight cultures of *E. coli *were adjusted to an OD of 0.01 at 600 nm (1 × 10^7 ^c.f.u/ml). Culture medium was replaced with 900 μl of pre-warmed DMEM-F12 in presence of 50 μl NHS or HIS (5%) and PTECs were infected with 100 μl of the bacterial suspension. Bacterial contact with host cells was increased by centrifugation of plates at 600 g for 5 minutes. After 3 hours of incubation at 37°C, bacteria bound to PTECs were measured by lysing cells with 1% Triton X-100 after vigorous washing to remove unattached bacteria. This would include internalised bacteria, but since binding exceeded internalisation by approximately 50 fold no correction was made. To assess the number of internalised bacteria, after 3 hours incubation PTECs were washed 3 times and then incubated for 1 hour in medium containing 100 μg/ml gentamicin to kill extra-cellular bacteria. Cells were then washed and lysed in 1% Triton X-100 in sterile H_2_O, and then plated on CLED agar plates (Oxoid, Basingstoke, UK). The agar plates were incubated at 37°C for 16 hours and the c.f.u counted. To investigate the involvement of type 1 fimbriae in the complement -dependent internalisation process, D-mannose or glucose was added to PTEC monolayers 20 minutes before bacteria were added and the internalisation assay carried out as above. In each experiment assays were performed in quadruplicate.

### Assessment of bacterial fimbrial adhesin expression

Expression of fimbriae was determined by haemagglutination of guinea pig (Harlan SeraLab, Loughborough, UK) or human erythrocytes in the presence and absence of mannose. Erythrocytes were prepared in 0.85% sodium chloride or 50 mM D-mannose in 0.85% sodium chloride (3% v/v). Bacterial cultures were centrifuged at 6,000 *g *for 6 minutes and resuspended to 1 × 10^10 ^cfu/ml in 0.85% sodium chloride. One hundred μl of *E. coli *suspension was added to an equal volume of erythrocyte solution on white tiles and gently rocked at room temperature for two minutes. Agglutination of guinea pig erythrocytes and the inhibition of agglutination in the presence of D-mannose confirmed the presence of type 1 fimbriae. P fimbriae were identified by agglutination of human erythrocytes that was not inhibited by addition of mannose.

### Detection of haemolysin production

To demonstration of haemolysin production bacteria were serially diluted 1 in 10 in PBS and 20 μl (about 2 × 10^6 ^bacteria) plated onto sheep blood agar (Oxoid). Plates were incubated for 16 hours at 37°C. Production of haemolysin was determined by haemolysis of the sheep erythrocytes producing a clear ring of agar around individual colonies.

### Presence of the CNF1 gene

CNF1 gene expression was determined by RT-PCR. The genomic DNA from *E. coli *strains was extracted using a quick alkaline lysis method [17]. A single colony was suspended in 25 μl of 0.5 N NaOH and incubated at room temperature for 30 minutes. 25 μl of 1 M HCl was added and the lysate diluted in 450 μl of sterile water, spun at 6,000 g for 6 minutes and the supernatant collected. PCR was carried out with 5 μl of lysate, 12.5 pmol each of primers (5'-CGCTTGGACTGGGGATAATT-3' and 5'-CTTCATAGTAGATGCCGCTC-3'), 200 μM of dNTPs, 3 U of GoTaq^® ^DNA Polymerase in a 25 μl of reaction buffer (Promega, Southampton, UK). The PCR cycle consisted of 40 seconds of denaturation at 94°C, 1 minute of primer annealing at 35°C, and 1 minute of extension/synthesis at 72°C. After 30 cycles of amplification, samples were incubated for another 10 minutes at 72°C. A 401 bp PCR products were visualised after electrophoresis on 1.2% agarose gel containing ethidium bromide.

### Serum sensitivity assay

Serum sensitivity was assessed according to the method of Miller and Robinson [18]. 10^8 ^cfu/ml of bacteria were washed and incubated in serially diluted NHS or HIS (in PBS) for 1 hour at 37°C. Samples of the bacterial suspension (50 μl of 1 in 10^5 ^dilutions) were plated onto agar plates. Serum resistance was determined by comparing the number of colonies from cultures incubated in NHS with those incubated in HIS. Serum resistance was defined as killing of less than 50% of organisms, intermediate resistance as killing of 50–99%, and serum sensitivity as > 99% of bacteria killed following incubation in up to 50% normal human serum.

### Statistical analysis

Bacteria binding and internalisation assays were performed in 4 replicate wells. Data from two separate experiments (total 8 wells) was pooled and analysed by students t-test for comparison of two variables, ANOVA with Bonferroni post test for multiple comparisons, Mann Whitney test or Fischer's exact test. P < 0.05 was regarded as significant.

## Results

### C3-dependent internalisation of E. coli isolates by PTECs

The ability of uro-epithelial cells to internalise bacteria has been recognised for some time. Our previous study suggested that the *E. coli *strain J96 can utilise C3 to increase internalisation into human PTECs. However it is still unknown whether this is a general feature of *E. coli*. Therefore, we determined whether C3-dependent internalisation by PTECs is seen with *E. coli *isolates from patients with acute UTI.

**Figure 1:**
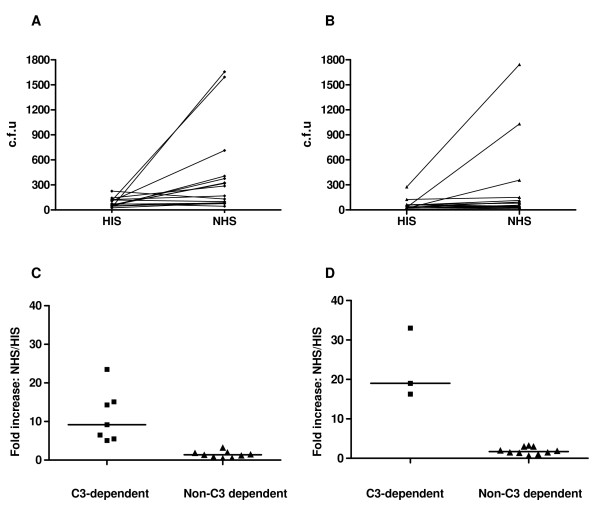
**Internalisation of PTECs by *E. coli *isolates**. 16 isolates of *E. coli *from the urine of patients with clinical UTI (**A **and **C**) and 15 isolated from blood cultures when the source was the urinary tract (**B **and **D**) were assessed to determine whether they demonstrated C3-dependent internalisation. (**A**) and (**B**) shown the number of bacteria internalised by PTECs in the presences of 5% NHS or HIS (mean of 4 separate wells per isolate). C3-dependent internalisation was arbitrarily defined as a 5-fold increase in the number of bacteria internalised in the presence of NHS compared with HIS. 7 urine isolates (43.75%) (**C**) and 3 (20%) blood isolates (**D**) demonstrated C3-dependent internalisation. The results were reproducible in two independent experiments.

16 *E. coli *isolates from the urine of patients with symptoms of acute lower UTI (Figure 1A) and 15 isolated from blood cultures (patients with simultaneous UTI) (Figure 1B) were assessed to determine whether they demonstrated C3-dependent internalisation. The number of intracellular bacteria was quantified after co-incubation of PTECs and *E. coli *isolates in the presence of 5% NHS or HIS. Only some *E. coli *isolates showed an increase in the number of intracellular bacteria after incubation with NHS (as a source of C3). The ratio of intracellular bacteria in the presence of NHS and HIS was used to assess the effect of complement on internalisation (8 replicate wells were used for each strain). C3-dependent internalisation was arbitrarily defined as a five-fold increase in the number of bacteria internalised in the presence of NHS compared with HIS. Using this criterion, 7 isolates from urine culture (44%, Figure 1C) and 3 isolates from blood (20%, Figure 1D) demonstrated C3-dependent internalisation. Therefore, by this criterion, C3-dependent internalisation is not a feature of all strains of *E. coli*, suggesting the requirement for a strain-dependent bacterial factor to act synergistically with complement opsonisation.

### The level of C3 opsonisation of E. coli isolates

**Figure 2:**
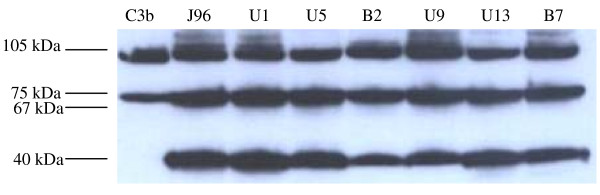
**C3 deposition on *E. coli *isolates**. 6 *E. coli *isolates (lane 3–8) were incubated with 5% NHS in culture medium for 30 minutes. C3 deposition was detected by Western blot analysis. Lane1, Purified C3b (0.1 μg); lane 2 J96 were incubated with 5% NHS; lanes 3–5 isolates showing positive for C3-dependent internalisation (U1, U5, B2); lanes 6–8, isolates not showing C3-dependent internalisation (U9, U13, B7). The presence of C3b is indicated by 105 kDa (α' chain) and 75 kDa (β chain) bands, iC3b by 75 kDa (β chain), 67 kDa (α1 chain), and 40 kDa (α2 chain) bands.

Opsonisation of the bacteria by C3 is critical for C3-dependent internalisation. Following activation, C3 is cleaved into C3a and C3b, exposing an internal thiolester bond allowing the C3b to bind covalently to hydroxyl groups (carbohydrates) or amine groups (proteins) on the pathogen surface. To determine the level of C3b deposition on the surface of the *E. coli *isolates, we performed C3 Western blotting using elute from isolates incubated with 5% NHS. The intensity of C3b was comparable in isolates irrespective of whether or not they demonstrated C3-dependent internalisation (Figure 2). Therefore, the differences in internalisation could not be explained by differences in the level of complement opsonisation.

### Virulence factors that lead to heterogeneity between E. coli isolates

Three broad classes of virulence factors have been identified in *E. coli *associated with UTI: adhesins, siderophores(aerobactin), and toxins. Other factors, such as capsules, lipopolysaccharide and serum sensitivity may also be important. Therefore, we examined the expression of these factors in the 31 *E. coli *isolates.

Table 1 shows the prevalence of virulence factors among the 16 urine *E. coli *isolates. Type 1 fimbriae were found in all (7/7) of the isolates demonstrating C3-dependent internalisation, whereas only two out of 9 strains that did not show C3-dependent internalisation had type 1 fimbriae (Table 2, P = 0.0032, Fischer's exact test). The presence of P fimbriae, expression of CNF1 or α-haemolysin were not associated with C3 dependent internalisation. As would be predicted from previous reports, there was a genetic linkage between the presence of cnf1^+ ^and the gene for α-haemolysin, hly^+^, in these isolates. But this did not associate with C3-dependent internalisation. Most strains studied were resistant to the lytic effects of complement (Table 1).

**Table 1:** Phenotyping of *E. coli *urine isolates.

Strains	Internalisation rate (NHS/HIS)	Type1- fimbriae	P-fimbriae	CNF1	Serum resistance	α-Haemolysin
J96	25	P	P	P	P	P
Internalised						
U1	9.2	P	P	P	P	P
U2	6.5	P	N	P	P	P
U3	14.3	P	N	P	P	N
U4	5.5	P	N	N	P	N
U5	5.1	P	N	N	P	N
U6	15.1	P	N	N	P	N
U7	23.5	P	N	N	P	N
Non-internalised						
U8	2.1	P	N	P	P	P
U9	0.55	P	N	P	P	P
U10	0.83	N	P	P	P	P
U11	1.5	N	N	N	P	N
U12	1.2	N	N	N	P	N
U13	1.9	N	N	N	P	N
U14	3.25	N	N	N	P	N
U15	1.375	N	N	N	N	N
U16	0.47	N	N	N	N	N

In 16 urine *E. coli *isolates, bacterial virulence factors (including type-1, P fimbriae, CNF1, serum resistance, and α-Haemolysin) were examined to determine their correlation to C3-dependent internalisation (P positive, N negative). All experiments were repeated at least three times.

**Table 2:** The association between virulence factors and C3-dependent internalisation in urine isolates.

Bacterial virulence factors	Strains demonstrating C3-dependent internalisation	Strains not demonstrating C3-dependent internalisation	Fischer's exact test
Type 1 fimbriae	7/7 (100%)	2/9 (22.2%)	P = 0.0032*
P fimbriae	1/7 (14.3%)	1/9 (11.1%)	nsd
CNF1	3/7 (42.9%)	3/9 (33.3%)	nsd
Serum resistance	7/7 (100%)	7/9 (77.8%)	nsd
Haemolysin	2/7 (28.6%)	3/9 (33.3%)	nsd

The strength of association between virulence factors and C3-dependent internalisation was determined using Fischer's exact test.

In fifteen blood isolates, type 1 fimbriae were also expressed by all of the isolates demonstrating C3-dependent internalisation (P = 0.0338, Fischer's exact test) (table 3 and 4). A greater proportion of blood isolates expressed invasion factors such as P fimbriae and α-haemolysin than urine isolates, as would be predicted from previous reports [19,20], however their presence did not correlate with C3-dependent internalisation.

**Table 3:** Phenotyping of *E. coli *blood isolates.

Strains	Internalisation rate (NHS/HIS)	Type 1- fimbriae	P- fimbriae	CNF1	Serum resistance	α-Haemolysin
J96	25	P	P	P	P	P
Internalised						
B1	6.3	P	P	P	P	P
B2	33	P	P	P	P	P
B3	19	P	N	N	P	N
Non-internalised						
B4	3.5	P	P	P	P	P
B5	3.3	P	P	N	P	P
B6	3.0	N	P	N	P	P
B7	1.2	N	P	N	P	P
B8	1.5	N	P	N	P	N
B9	2	N	P	N	P	N
B10	1.2	N	P	N	P	N
B11	0.7	N	N	P	P	P
B12	1.3	N	N	N	P	P
B13	1	N	N	N	P	N
B14	0.5	N	N	N	P	N
B15	2.2	N	N	N	P	N

In 15 blood isolates, bacterial virulence factors (including type-1, P fimbriae, CNF1, serum resistance, and α-Haemolysin) were examined to determine their correlation to C3-dependent internalisation (P positive, N negative). All experiments were repeated at least three times.

**Table 4:** The association between virulence factors and C3-dependent internalisation in blood isolates.

Bacterial virulence factors	Strains demonstrating C3 -dependent internalisation	Strains not demonstrating C3-dependent internalisation	Fischer's exact test
Type 1 fimbriae	3/3 (100%)	2/12 (16.7%)	P = 0.0338
P fimbriae	2/3 (66.7%)	7/12 (58.3%)	nsd
CNF1	2/3 (66.7%)	2/12 (16.7%)	nsd
Serum resistance	3/3 (100%)	12/12 (100%)	nsd
Haemolysin	2/3 (66.7%)	6/12 (50.0%)	nsd

The strength of association between virulence factors and C3-dependent internalisation in blood isolates was determined using Fischer's exact test.

### Effects of mannose on bacterial binding and C3-dependent internalisation

**Figure 3:**
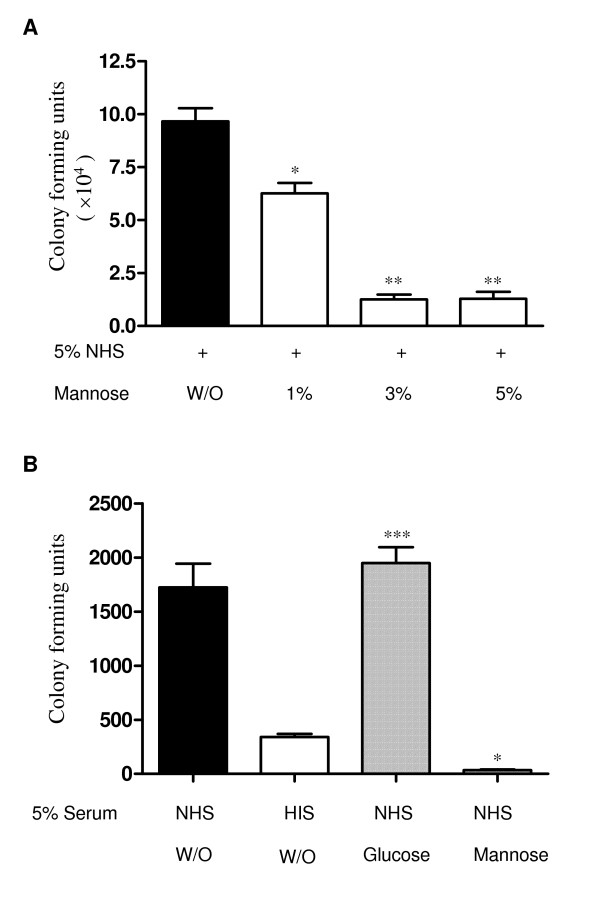
**Mannose prevents type 1 fimbriated *E. coli *binding to and invasion of PTECs**. (A) Binding of type 1 fimbriated *E. coli *(J96) to PTECs was assessed in the presence or absence of mannose. Mannose was added to the cells 30 minutes before the addition of bacteria and serum. Mannose prevents type 1 fimbriae-mediated binding in a concentration-dependent manner (> 80% inhibition in the presence of 3% mannose). P values are for comparisons between the absence and presence of mannose. * P < 0.005, **, P < 0.001. (B) Internalisation of type 1 fimbriated *E. coli *(J96) by PTEC was assessed in the presence of either mannose or glucose. 3% mannose or glucose was added to the cells 30 minutes before the addition of bacteria and serum. The presence of mannose significantly reduced the rate of bacterial internalisation (***, P < 0.0001 compared with Glucose). The results are representative of 3 separate experiments. Mean+/- SEM, n = 3 per experiment.

Previous studies have shown that type 1 fimbriae alone can mediate pathogen adherence to host epithelium and induce pathogen internalisation [9]. Mannose can prevent type 1 fimbriae-mediated bacterial adherence to uroepithelial cells. Therefore, we used mannose blockade to study the interaction between type 1 fimbriae-mediated bacterial adherence/internalisation and C3 opsonisation. Assessment of bacterial binding showed that the presence of mannose in culture medium inhibited type 1 fimbriae-mediated J96 binding to PTECs in a dose dependent manner (Figure 3A). 3% mannose also reduced C3-dependent internalisation by PTECs. In contrast the same concentration of glucose had no effect on bacterial internalisation (Figure 3B). Therefore, blocking type 1 fimbriae-mediated binding can efficiently inhibit C3-dependent internalisation.

### FimH mediates opsonised E. coli adherence and invasion of PTECs

**Figure 4:**
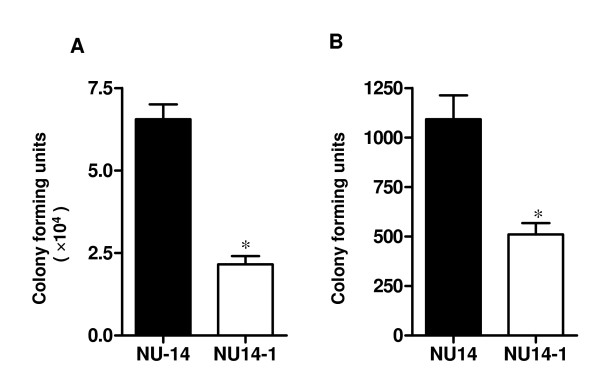
**FimH mediates opsonised *E. coli *adherence and invasion of PTECs**. Adherence assays (A) and internalisation assays (B) were performed in the presence of 5% NHS. The type 1 fimbriated *E. coli *cystitis isolates, NU14 is more efficient at adhering to and internalisation into PTEC than the isogenic fimH^- ^mutant, NU14-1 (*, P < 0.005). Data are shown as Mean ± SEM [n = 3 (for adherence assay) or n = 4 for (internalisation assays)], a representative of three independent experiments.

FimH mutation provided another means of blocking type 1 fimbriae-mediated bacterial adherence and internalisation of human PTECs. Type 1-fimbriated cystitis isolate, NU14 or the isogenic Fim H^- ^mutant NU14-1 were co-cultured with PTECs in the presence of 5% NHS. As shown in Figure 4, a significant reduction in the number of bacteria bound to and internalised by PTECs were seen in FimH^- ^mutant strain compared to the type 1 fimbriated wild type strain (Figure 4). This result further confirmed that type 1 fimbriae-mediated binding is required for C3-dependent internalisation.

## Discussion

Whether or not complement opsonisation increased internalisation into renal epithelial cells was assessed for 16 *E. coli *isolates from the urine of patients with cystitis and 15 isolated from blood cultures taken from patients with simultaneous UTI. Not all *E. coli *isolates demonstrated C3-dependent internalisation (taken arbitrarily as a five-fold increase in bacterial internalisation in the presence of a source of complement). This was only evident in 44% of urinary and 20% of blood isolates. Complement proteins are present in the urine and their concentration increases significantly in the presence of urinary tract infection, sufficient to opsonise bacteria [13,14]. Therefore isolates of *E. coli *which were internalised more efficiently when opsonised may be able to gain access to a favourable intracellular niche, protected from immune attack and antibiotic treatment. Whether these isolates are more likely to cause persistent or recurrent infection has not been addressed in this current study.

We next investigated whether there was an association between a specific bacterial phenotype and increased internalisation when opsonised with complement. All strains that demonstrated C3-dependent internalisation also expressed type 1 fimbriae, suggesting that there is co-operation between C3 and type 1 fimbriae to achieve maximal bacterial internalisation. To confirm the importance of type 1 fimbriae, internalisation was assessed in the presence of excess mannose to prevent type 1 fimbriae-mediated binding to epithelial cells. Only very low levels of internalisation were seen under these conditions, even in the presence of complement opsonisation. Therefore, type 1 fimbriae-mediated binding is an absolute requirement for internalisation irrespective of C3 opsonisation. In addition, a deletion of the FimH adhesin significantly abrogated binding and intracellular invasion of opsonised *E. coli*, further confirming that type 1 fimbriation is required for C3-dependent internalisation.

We could not demonstrate a role for P fimbriae in intra-cellular invasion by *E. coli*. P fimbriae, and specifically the class II PapG adhesin, are clinically associated with acute pyelonephritis in humans. They bind to Gal(α1-4)Galβ moieties present in membrane glycolipids of the human kidney [21]. Fimbrial transformation and mutant studies have suggested P fimbriae can enhance bacterial binding and induce a robust inflammatory response during the early kidney colonisation [22]. However, consistent with our present data, a previous study on bladder cells suggested that adherence mediated by the PapG did not result bacteria internalisation [9].

Notably, the percentage of isolates expressing type 1 fimbriae is much lower in bacteraemia isolates than in urinary isolates (33% versus 56%). In contrast a higher percentage expressing P fimbriae was seen (60% versus 12.5%) in bacteraemia isolates. It is likely that 'crosstalk' occurs between the regulators of the different fimbrial systems in pathogenic *E. coli*. Classically pyelonephritis strains are more likely to contain and express P fimbrial gene clusters and therefore down-regulate type 1 fimbriae expression [23]. This may explain the different patterns of clinical infection caused by different strains of *E. coli*.

## Conclusion

**Figure 5:**
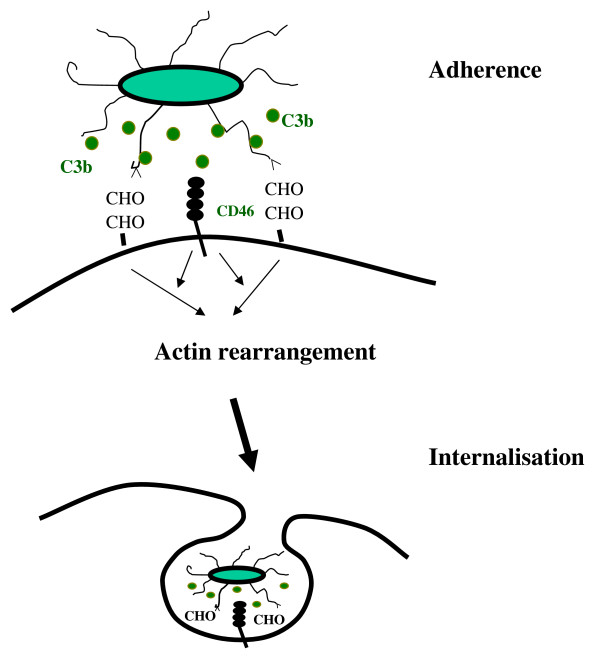
**Diagram showing possible involvement of both CD46 and type 1 fimbrial receptor signalling in the internalisation of *E. coli *by PTECs**. Internalisation of *E. coli *is initiated by type 1 fimberiae mediated adhesion to epithelium mannosylated glycoproteins receptor. This may be sufficient to induce internalisation alone. However, during UTI, *E. coli *can be opsonised by urine C3 in urinary tract space. C3b bound on bacteria surface interact with cell surface expressed CD46. This C3b-CD46 interaction could activate host cells and augments the direct interaction of fimH with manosylated receptor resulting in a high internalisation. Inhibition and FimH mutant experiments indicate that non-opsonic interactions are necessary for *E. coli *adherence to and invasion of PTECs.

Type 1 fimbriae mediated-binding is essential for C3-dependent internalisation. We do not know whether this is a co-operative, synergistic action or the additive activities of two factors. Since, FimH alone can mediate intra-cellular invasion, we suggest that the C3 opsonisation augments the signalling initiated by FimH-mediated binding (Figure 5). Studies to analyse the mechanism by which C3 receptor(s) (CD46) and the receptors for FimH interact are important to fully understand invasion of human urinary tract by pathogenic *E. coli*.

## Authors' contributions

NSS and SHS conceived of the study. NSS and KL designed the experiments and wrote the paper. KL, HY and WZ performed experiments and analysed data. WZ and SHS helped with research design and manuscript discussion. All authors have read and approved the final manuscript.
